# VExUS in Mechanical Circulatory Support: Unveiling the Role of Doppler Ultrasound in Assessing Venous Congestion

**DOI:** 10.24908/pocusj.v11i01.19348

**Published:** 2026-04-22

**Authors:** Abhilash Koratala, Mark Jacobs, Uday Gulati, Ayush Sutaria, Ali A Naqvi, Anthony Carlese

**Affiliations:** 1 Division of Critical Care Medicine, Montefiore Medical Center, New York, NY, USA; 2 Division of Nephrology, Medical College of Wisconsin, Milwaukee, WI, USA

**Keywords:** Venous congestion, ECMO, Ballon pump, Doppler

## Abstract

Persistent congestion in decompensated heart failure predicts poor outcomes. The Venous Excess Ultrasound (VExUS) score, which integrates hepatic, portal, and intrarenal Doppler waveforms, provides a dynamic, noninvasive measure of venous congestion and outperforms right atrial pressure alone. Its utility during mechanical circulatory support remains largely unexplored. We report a case of right ventricular failure requiring venoarterial extracorporeal membrane oxygenation (VA ECMO) and intra-aortic balloon pump (IABP). Despite changes in arterial waveform with IABP settings, venous Doppler signals remained stable, suggesting minimal impact on congestion. In contrast, clamping the ECMO circuit led to marked worsening of portal and intrarenal waveforms, highlighting the drainage cannula's role in right-sided offloading. Hepatic vein Doppler remained unchanged due to severe tricuspid regurgitation (TR). This case illustrates the value of VExUS in monitoring venous congestion during advanced cardiac support and its potential in guiding decongestion strategies, weaning decisions, and right heart support. Further research is needed to validate these observations.

## Introduction

**Figure 1.**
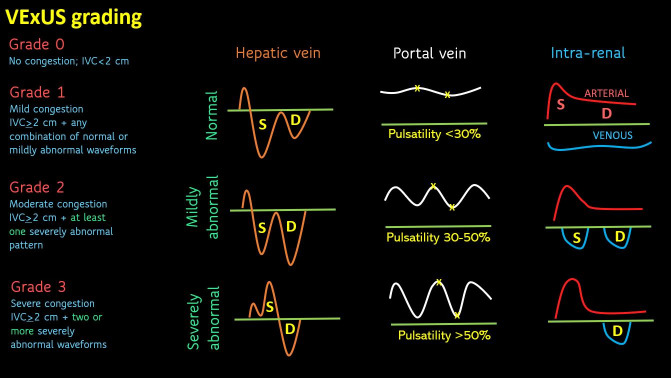
Venous Excess Ultrasound (VExUS) grading system to quantify systemic venous congestion. Briefly, when the inferior vena cava (IVC) diameter exceeds 2.0 cm, congestion is graded by Doppler abnormalities: systolic wave (S)/diastolic wave (D) ratio <1.0 with the S wave below baseline indicates mild hepatic vein congestion, while an S wave above baseline indicates severe congestion. Portal vein pulsatility of 30–50% denotes mild abnormality, while >50% indicates severe congestion (measured over one cardiac cycle). In the intrarenal vein, distinct S and D waves indicate mild congestion, whereas a D-wave-only pattern signifies severe congestion. Intrarenal vein congestion can also be quantified using flow interruptions or the stasis index, where larger flow gaps indicate more severe congestion. Figure reused with permission from NephroPOCUS.com.

Persistent congestion in decompensated heart failure predicts worse outcomes. Doppler ultrasonography provides real-time assessment of venous flow alterations indicative of congestion and has recently garnered increasing attention. The Venous Excess Ultrasound (VExUS) score, introduced in 2020 from cardiac surgery data, quantifies venous congestion using hepatic, portal, and intrarenal vein Doppler [1]. Figure 1 illustrates the grading system. Given that congestion results from the interplay of fluid overload, elevated right atrial pressure (RAP), and venous compliance, VExUS provides better predictive value for congestive organ injury compared to RAP alone [2]. These waveforms also change dynamically with treatment, making them useful for ongoing monitoring. Recent evidence suggests that the VExUS score enhances the prediction of in-hospital mortality in heart failure patients compared to conventional models [3]. In patients with severe tricuspid regurgitation (TR), hepatic vein systolic reversal may persist despite decongestion, which is considered a limitation of VExUS. Portal vein flow patterns still improve in these cases, which serve as a progress marker [4]. However, data on VExUS in mechanical circulatory support, particularly venoarterial extracorporeal membrane oxygenation (VA ECMO), is lacking. This case file highlights the effects of VA ECMO and intra-aortic balloon pump (IABP) on venous waveforms. To ensure consistency, all images were acquired by a single operator (author AK), who has performed over 500 documented VExUS studies. All images were also taken using the same ultrasound machine (Philips 5500P).

## Case Presentation

Briefly, this case describes a 50-year-old man with a history of aortic root aneurysm repair and coronary re-implantation, who developed intraoperative right ventricular dysfunction, likely due to myocardial infarction. He underwent single-vessel coronary artery bypass grafting (saphenous vein graft to the right coronary artery) with IABP placement. However, he deteriorated despite dual inotropes (dobutamine, epinephrine) and was cannulated for VA ECMO three days later. The patient also developed acute kidney injury that required continuous renal replacement therapy.

The images were obtained while the patient was receiving both IABP and VA ECMO support. The IABP was set to a 1:1 configuration. At the time, pulmonary artery catheter measurements demonstrated a central venous pressure (CVP) of 16 mmHg and pulmonary artery pressures (PAP) of 31/20 mmHg. The ECMO circuit configuration was right femoral vein drainage and left femoral artery return, with a flow rate of 3 L/min. Transthoracic echocardiography revealed severe right ventricular (RV) dysfunction and qualitatively severe TR, though imaging was limited by suboptimal acoustic windows (Supplementary Material S1). The left ventricular systolic function was mildly reduced, with regional wall motion abnormalities in the right coronary artery territory.

**Figure 2.**
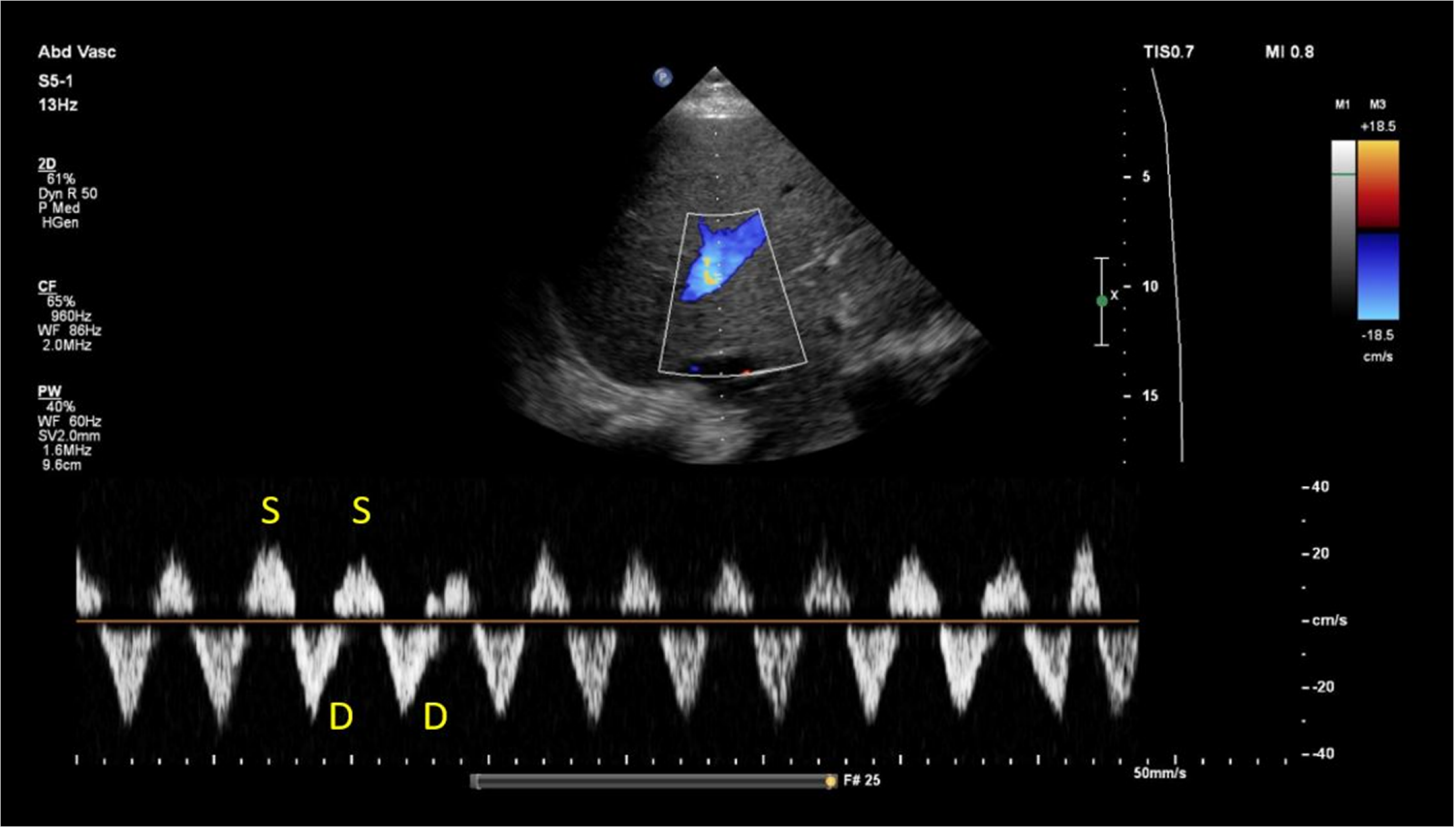
Hepatic vein Doppler demonstrating systolic flow reversal.

The inferior vena cava (IVC) appeared plethoric, measuring >2.1 cm in diameter. Supplementary Material S2 demonstrates a right upper quadrant lateral view, revealing the ECMO drainage cannula within the IVC and the intra-aortic balloon pump positioned in the abdominal aorta. Hepatic vein Doppler demonstrated systolic flow reversal, indicative of severe venous congestion (Figure 2). Although a built-in electrocardiogram was not available on the ultrasound machine to definitively identify systolic and diastolic waves, interpretation was supported by the concurrently recorded CVP waveform from the pulmonary artery catheter, which showed a prominent y descent. Additionally, impaired tricuspid annular excursion along with severe TR likely contributed to the reversal of hepatic vein systolic flow.

**Figure 3.**
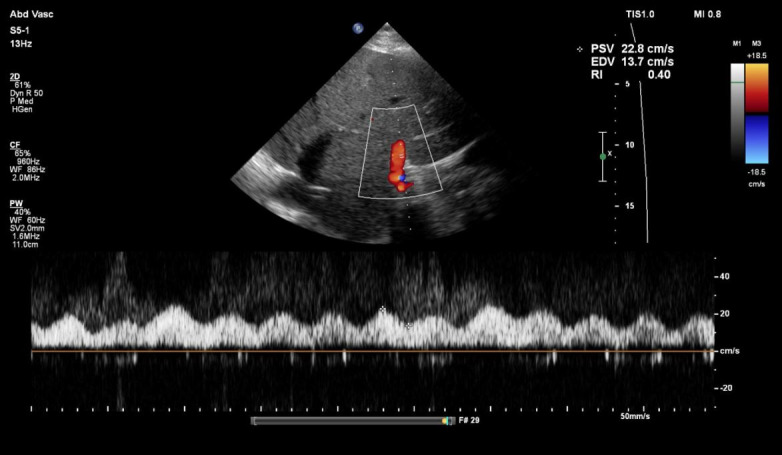
Portal vein Doppler demonstrating mildly increased pulsatility.

**Figure 4.**
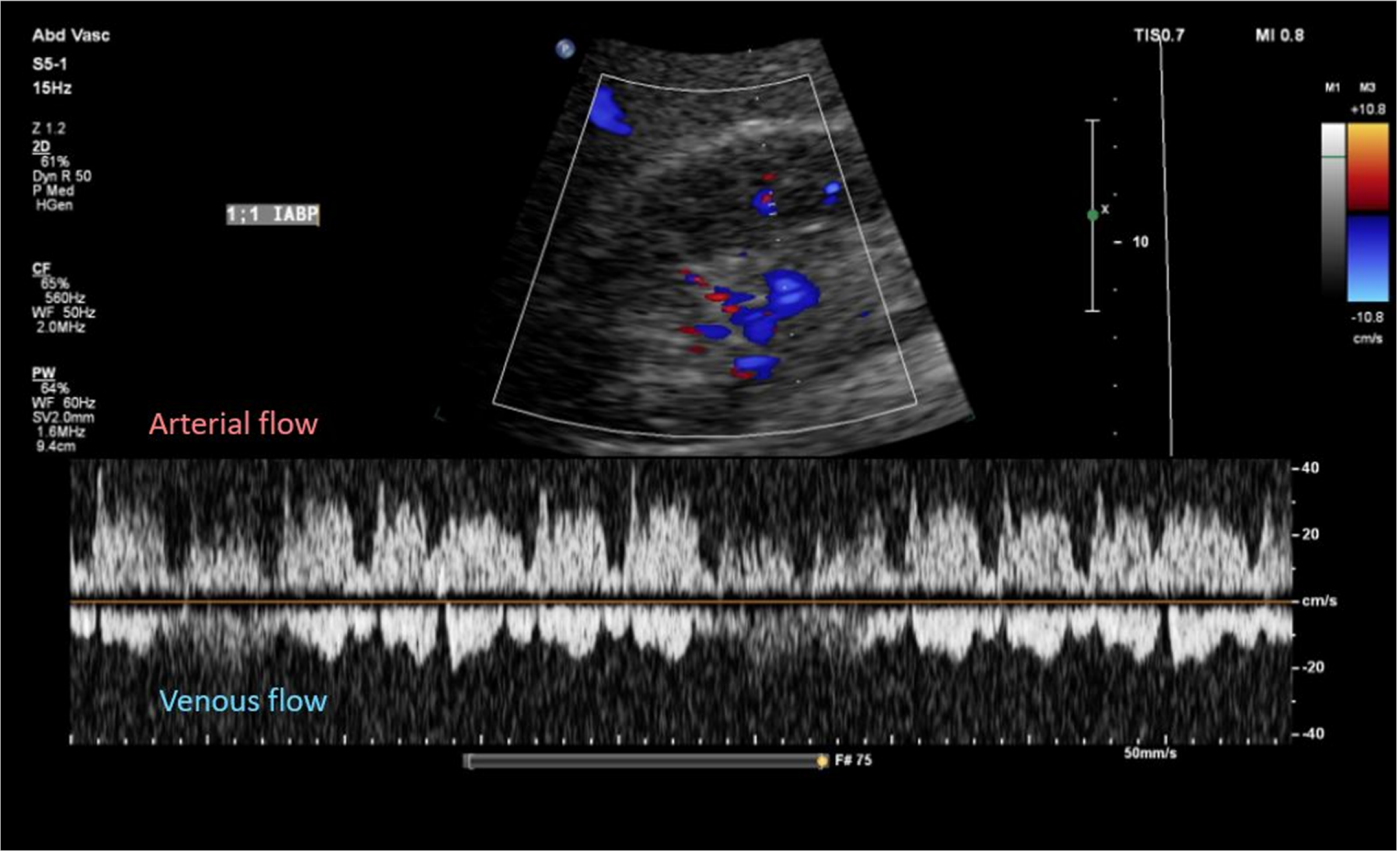
Intrarenal Doppler showing minor flow interruptions in the vein.

Portal vein Doppler exhibited mild pulsatility, with a pulsatility fraction of approximately 40%. Intrarenal venous Doppler demonstrated mildly pulsatile flow with minor flow interruptions (Figures 3 and 4).

**Figure 5.**
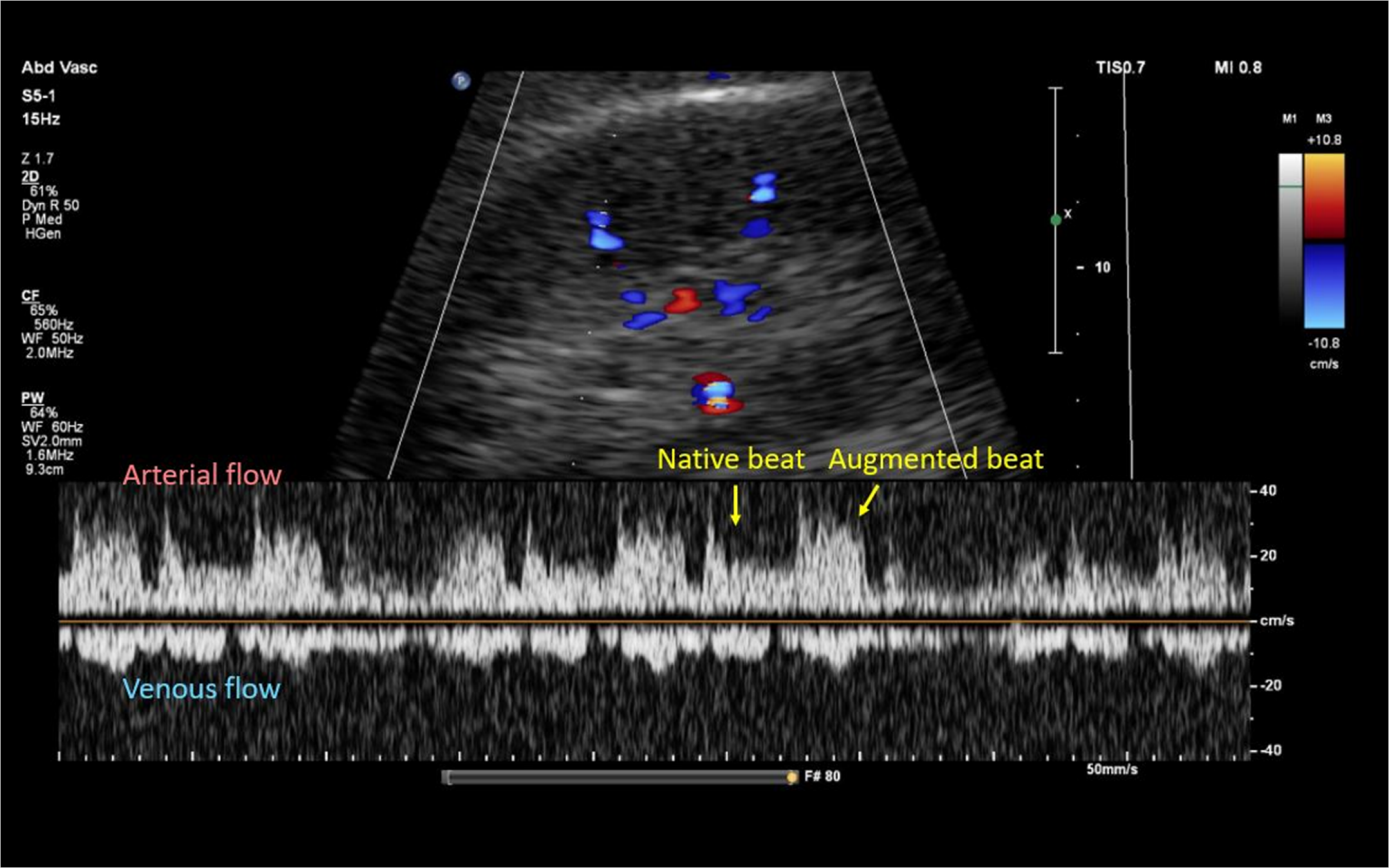
Intrarenal Doppler at intra-aortic balloon pump (IABP) frequency of 2:1.

**Figure 6.**
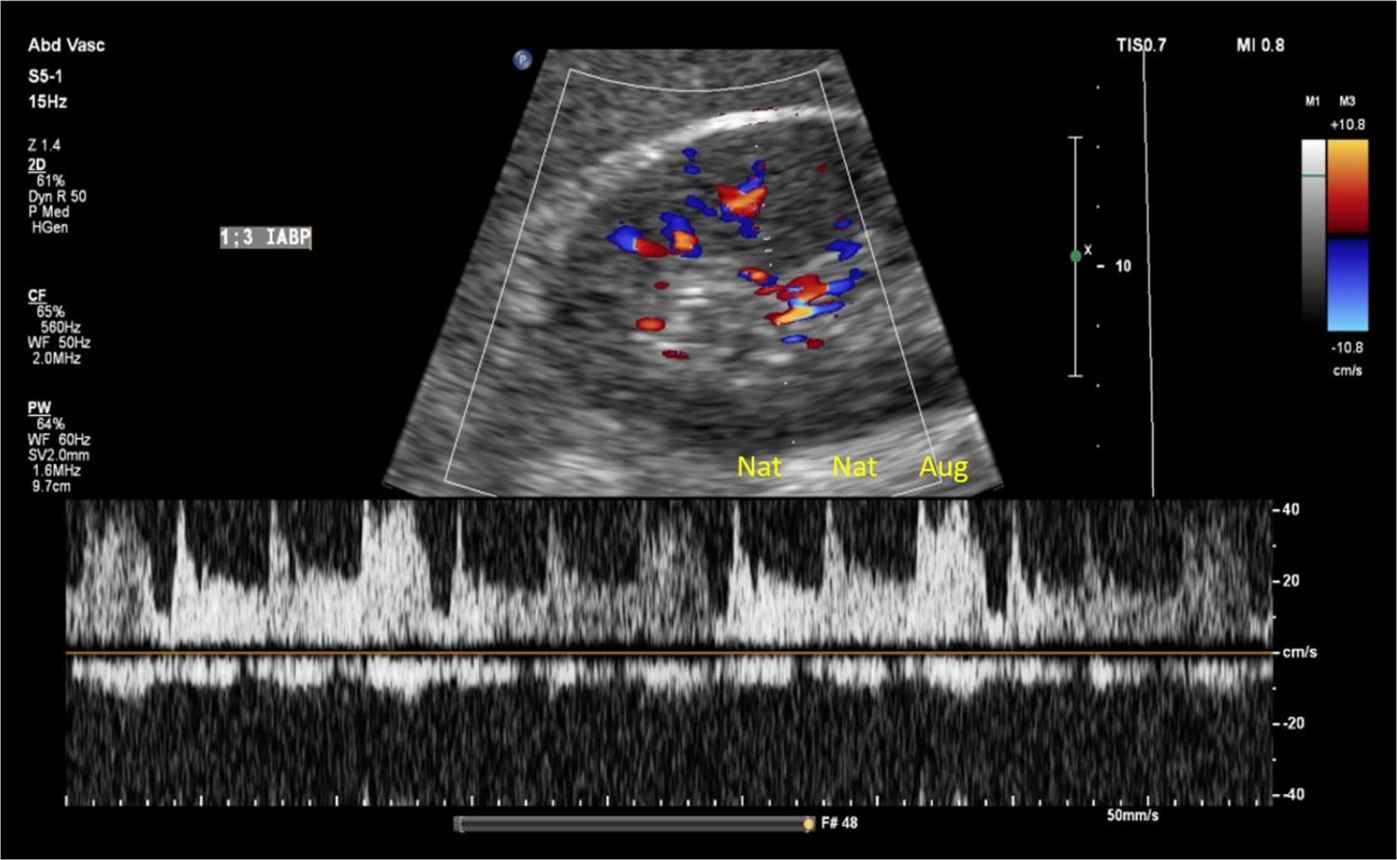
Intrarenal Doppler at intra-aortic balloon pump (IABP) frequency of 3:1. Nat, native arterial beat; Aug, balloon augmented flow.

**Figure 7.**
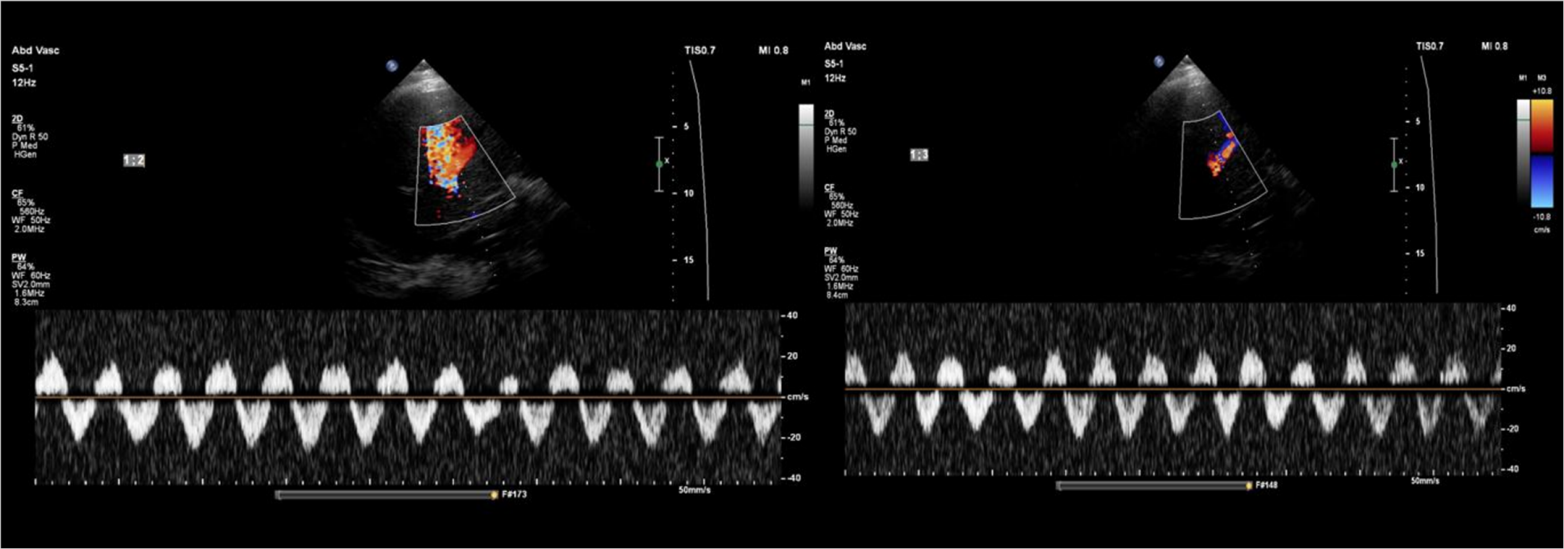
Unchanged hepatic vein flow with intra-aortic balloon pump (IABP) frequency.

Adjusting the IABP operational mode to 1:2 and 1:3 frequency resulted in no significant changes to the intrarenal venous flow, despite clear alterations in the arterial waveform reflecting native and augmented flow. Similarly, no appreciable changes were observed in the hepatic vein waveform (Figures 5-7).

**Figure 8.**
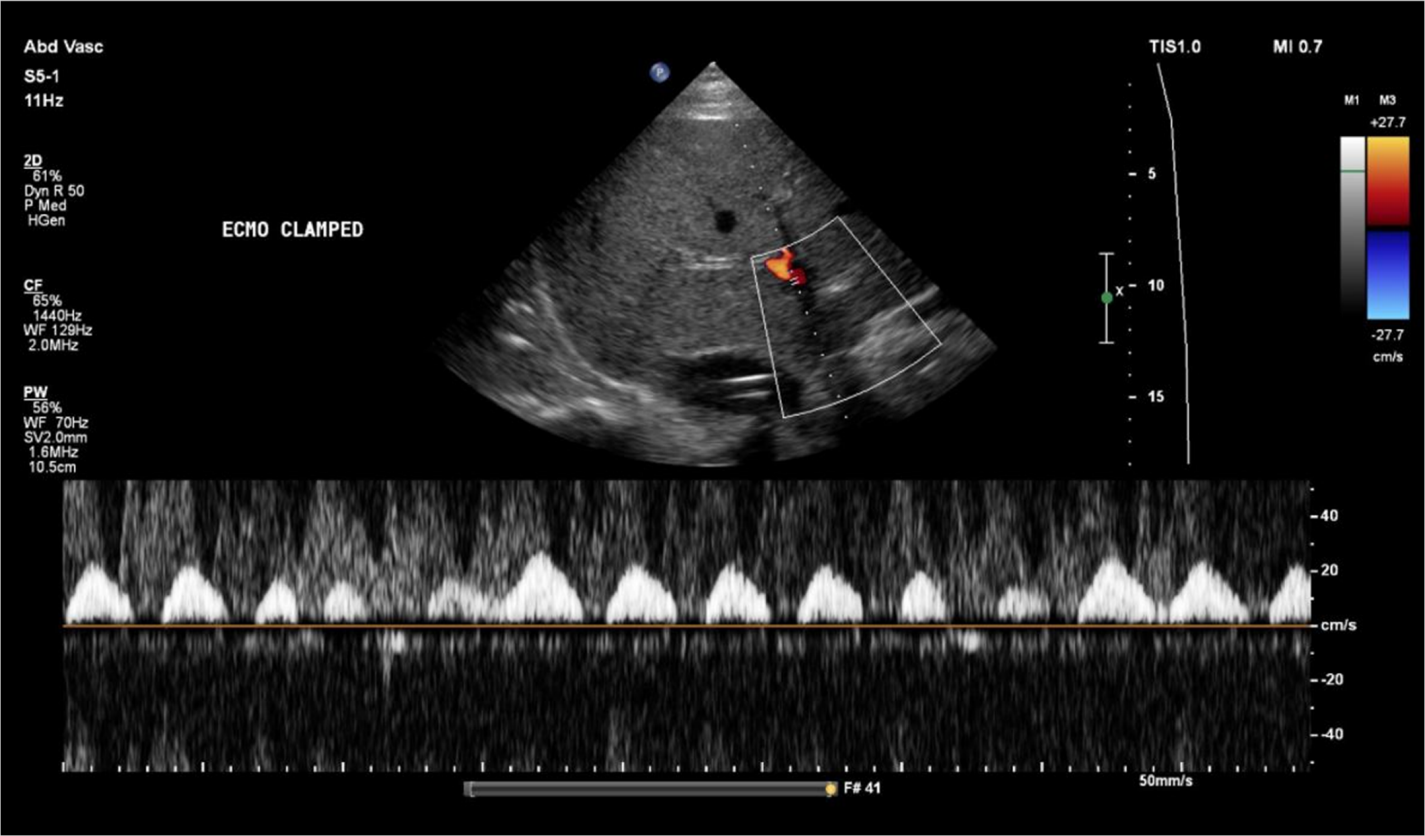
Increased portal vein pulsatility following extracorporeal membrane oxygenation (ECMO) clamping.

**Figure 9.**
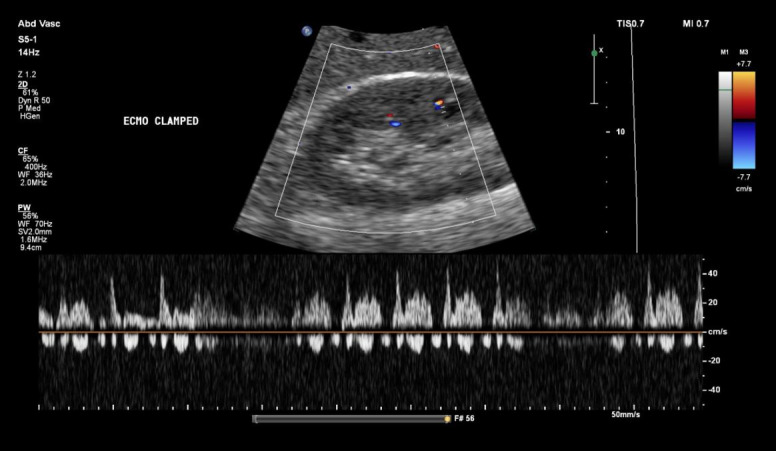
Increased interruptions in intrarenal venous flow following extracorporeal membrane oxygenation (ECMO) clamping.

**Figure 10.**
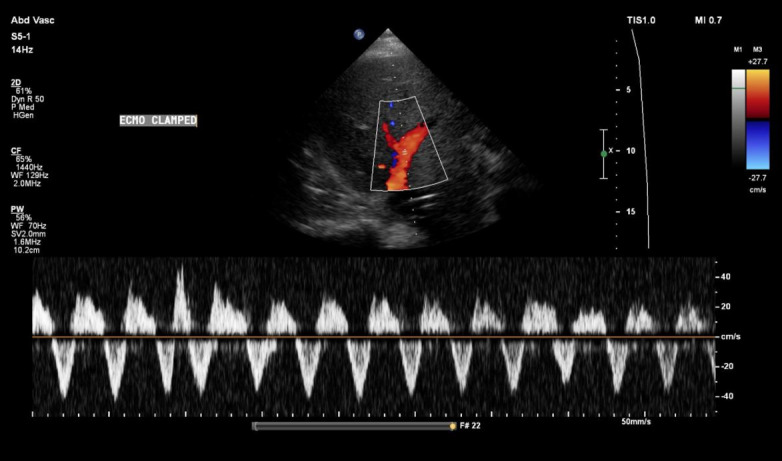
No change in hepatic vein flow after extracorporeal membrane oxygenation (ECMO) clamping.

However, clamping of the ECMO circuit led to a marked deterioration in venous congestion. Portal vein pulsatility increased to 100%, with pronounced flow interruptions (Figure 8). A similar increase in flow interruptions was observed in the intrarenal venous Doppler, consistent with worsening systemic congestion (Figure 9). Hepatic vein flow remained unchanged, likely because it was already at its most severe state (Figure 10). These findings were accompanied by a modest increase in CVP of 3 mmHg and a rise in PAP to 34/23 mmHg, as measured by the pulmonary artery catheter. Of note, clamping was carried ut by a trained perfusionist in the presence of a cardiac intensivist, with careful attention to safety and appropriate duration.

## Discussion

While recognizing the limitations of a single case study and the operator-dependent nature of image acquisition, this case provides a few key insights that may have future research implications:

While IABP altered arterial flow dynamics, it did not appear to have a significant effect on venous congestion as assessed by Doppler ultrasound. This suggests that IABP and other LV venting strategies may have limited immediate impact on right-sided venous congestion.

The VA ECMO drainage cannula effectively offloaded the right atrium. This reduced venous congestion despite persistent RV failure, while clamping led to an instantaneous worsening of congestion. This finding may help predict the risk of congestive organ injury post-decannulation, guide the need for mechanical right heart support, and assess ECMO weanability.

The dynamic nature of venous Doppler waveforms enables real-time monitoring of treatment response, offering additive value to invasive pressure measurements, which are prone to errors from transducer positioning and zeroing. Notably, while hepatic vein Doppler may be affected by impaired RV excursion and TR—making the pursuit of a normal waveform potentially unsafe in this context—portal and intrarenal venous Doppler waveforms remain reliable indicators of systemic venous congestion. These findings open new avenues for further research in this area.
